# Use of task-shifting to rapidly scale-up HIV treatment services: experiences from Lusaka, Zambia

**DOI:** 10.1186/1472-6963-9-5

**Published:** 2009-01-09

**Authors:** Mary B Morris, Bushimbwa Tambatamba Chapula, Benjamin H Chi, Albert Mwango, Harmony F Chi, Joyce Mwanza, Handson Manda, Carolyn Bolton, Debra S Pankratz, Jeffrey SA Stringer, Stewart E Reid

**Affiliations:** 1 Centre for Infectious Disease Research in Zambia; Lusaka, Zambia; 2 Schools of Medicine and Public Health, University of Alabama; Birmingham, AL, USA; 3 Lusaka District Health Management Team; Lusaka, Zambia; 4 Zambian Ministry of Health; Lusaka, Zambia

## Abstract

The World Health Organization advocates task-shifting, the process of delegating clinical care functions from more specialized to less specialized health workers, as a strategy to achieve the United Nations Millennium Development Goals. However, there is a dearth of literature describing task shifting in sub-Saharan Africa, where services for antiretroviral therapy (ART) have scaled up rapidly in the face of generalized human resource crises. As part of ART services expansion in Lusaka, Zambia, we implemented a comprehensive task-shifting program among existing health providers and community-based workers. Training begins with didactic sessions targeting specialized skill sets. This is followed by an intensive period of practical mentorship, where providers are paired with trainers before working independently. We provide on-going quality assessment using key indicators of clinical care quality at each site. Program performance is reviewed with clinic-based staff quarterly. When problems are identified, clinic staff members design and implement specific interventions to address targeted areas. From 2005 to 2007, we trained 516 health providers in adult HIV treatment; 270 in pediatric HIV treatment; 341 in adherence counseling; 91 in a specialty nurse "triage" course, and 93 in an intensive clinical mentorship program. On-going quality assessment demonstrated improvement across clinical care quality indicators, despite rapidly growing patient volumes. Our task-shifting strategy was designed to address current health care worker needs and to sustain ART scale-up activities. While this approach has been successful, long-term solutions to the human resource crisis are also urgently needed to expand the number of providers and to slow staff migration out of the region.

## Background

Like many neighboring countries in sub-Saharan Africa, services for antiretroviral therapy (ART) in Zambia have expanded rapidly in recent years [1,2]. At the start of 2008, it was estimated that more than 156,000 HIV-infected adults and children had initiated HIV treatment, a forty-fold increase in less than five years. This progress has been made in spite of significant shortages of health care workers. Staffing deficits over this period were estimated at over 70% for doctors, clinical officers, and nurses [3]. With as many as 330,000 Zambians still in urgent need of HIV treatment country-wide, and more becoming eligible each year, successful and continued expansion of ART services will largely depend on the ability to address the growing human resource crisis. "Task-shifting" is the process of delegating tasks from more specialized to less specialized health workers and has been proposed as one of several possible solutions to the dire human resource shortages facing the African health sector [4].

To address its severe human resource shortage, the Zambian Ministry of Health has strongly supported an integrated program of task-shifting among providers [5]. Appropriate health care responsibilities have been transferred from physicians to mid-level clinicians (e.g., nurses and clinical officers) and from nurses to community health workers. The success of these programs has maximized the potential of health care providers, and allowed the continued expansion of services in the face of severe resource constraint. In this report, we describe our field experiences with task shifting in Lusaka, Zambia, where a large public-sector ART program has enrolled over 71,000 HIV-infected adults and children across 19 program sites [1,2]. We advocate a comprehensive, three-pronged approach to task-shifting that comprises training, on-site clinical mentoring, and continuous quality assurance. A structured approach is important so that clinical care is not compromised when clinical duties are initially shifted to less specialized health professionals. In fact, we have shown that improvements in clinical care quality indicators may be possible, even in the context of rapidly increasing patient volumes.

## Methods

### Provider roles in the task-shifting model

**Figure 1:**
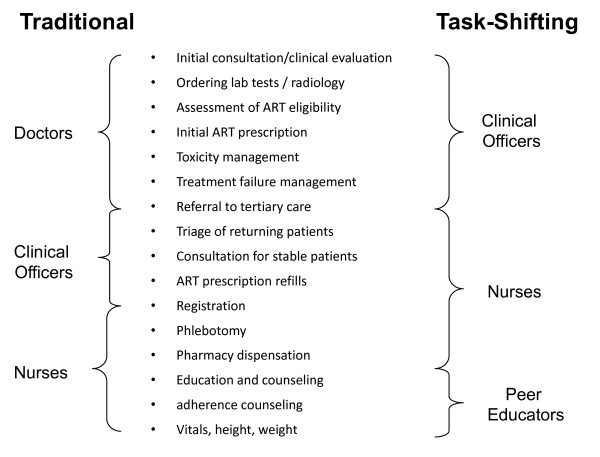
**Clinical care and administrative responsibilities in the "traditional" health care model in Zambia and the task-shifting model that has been introduced**.

The task-shifting model requires the transfer of specific clinical responsibilities to other providers who can be trained for the task (Figure 1). This approach has been possible in Lusaka because of the relative abundance of mid-level to lower level health providers compared to trained physicians, a scenario common to many African health systems. Task-shifting strategies often arise informally at the clinic level, where providers unofficially take on additional administrative and clinical responsibilities in order to ensure continued provision of services. In the setting of rapid ART expansion, however, we advocate a more structured cadre approach to ensure the provision of quality care. This is important since provision of HIV services is relatively new to many settings, requires longitudinal rather than acute and episodic care, and may grow increasingly complex with long-term drug toxicities, treatment failure recognition, and regimen changes. For an effective task-shifting approach, responsibilities for each cadre of health provider were identified, so that targeted training programs could be implemented.

Clinical officers (equivalent to nurse practitioners in North America and Europe) provide the majority of first-line health care and thus play a critical role in task-shifting and expanding access to HIV care. The traditional role of a clinical officer in our setting is to assess patients' eligibility for and prescribe antiretroviral drugs, and to identify toxicities and opportunistic infections. They have traditionally handled only routine cases, referring more complicated clinical problems to medical officers (i.e. doctors). As services have expanded, however, clinical responsibilities among clinical officers have increased owing to the local shortage of medical officers. To ensure that this task-shifting results in quality clinical care, we have focused on improving physical examination skills and clinical decision making.

The traditional role of nurses in Lusaka clinics focused on duties such as basic history-taking, vital signs measurement, adherence counseling, phlebotomy, and medication dispensation. Much of their time was spent in clerical duties and many of their tasks were administrative in nature. The focus of task shifting for nurses has been to develop their skills in screening and basic clinical assessment so that they may reduce the work burden of the clinical officers. Nurses are now trained to triage patients in waiting areas, direct patient flow, and manage stable patients in long-term HIV care.

In our programs, community health workers file patient charts, organize support groups, and perform home visits for patients with missed appointments. We have expanded the responsibilities of community health workers by establishing a new cadre called "peer educators." More experienced community health workers may apply for selection as peer educators through competitive interview process conducted regularly at each site. While not a requirement, nearly all candidates are HIV-infected patients who may or may not be on ART. Following intensive training, they perform administrative and basic clinical duties that are part of comprehensive patient care. Their main role is to conduct adherence counseling and health education for patients. This task is critical but time-consuming; use of peer educators frees nurses to perform more clinical duties. Peer educators may also assist nurses by recording patient height and weight, and assessing vital signs such as temperature, blood pressure and heart rate. They are provided a monthly stipend in accordance with district pay scales; this is in contrast to community health workers, who are considered volunteers and receive only meal and transport allowances.

### Training

Didactic training is a cornerstone of the Zambian Ministry of Health's curriculum for HIV care and treatment. Courses vary from one to three weeks in length and provide in-depth coverage of topics such as adult HIV care, pediatric HIV care, and adherence counseling. These are requirements for nearly all health staff providing HIV-related health services.

With support from the Ministry of Health, we have included targeted trainings focused on task-shifting. Experienced HIV nurses undergo triage training, to ensure that all patients are assessed and prioritized for care based on clinical need. Triage training focuses on the following skills: (1) patient assessment and management of urgent problems, (2) recognition of toxicities and severe illness, and (3) interpretation of laboratory investigations. Nurses then undergo advanced training on care of the stable HIV patient, similar to the Integrated Management of Adult and Adolescent Illness (IMAI) program. This five-day training focuses on clinical evaluation and World Health Organization HIV disease staging; opportunistic infection prophylaxis and treatment; antiretroviral therapy eligibility; and antiretroviral drug toxicity and management. A key focus is on the early recognition of signs and symptoms requiring referral to a higher level.

Peer educators are trained to provide health education, counsel patients on adherence and other HIV related issues, and perform basic health worker duties, such as recording patient demographic information, heights and weights, and basic vital signs using digital equipment. They receive a three-week classroom style training that focuses on communication skills, adherence and behavior change counseling, confidentiality, disclosure, HIV transmission prevention, and basic HIV care and treatment. Pediatric peer educators are trained to provide counseling services for children and assist families with disclosure counseling.

### Mentoring

Didactic training is too brief to result in sustained changes in clinical practice. After formal implementation of clinic systems, mentors continue to work at the site level to build problem-solving capacity and leadership. A core staff of local physicians, clinical officers, and nurses participate in on-site weekly clinical teaching rounds, and provide one-on-one mentoring specific to their cadre. HIV clinicians from the United States, Canada, and South Africa make regular visits, and are consulted via internet for complicated cases. This system facilitates continuous professional development for all cadres of health staff in the continuously evolving field of HIV medicine.

Tailored programs have also been created for each targeted cadre of health care provider. In order to develop physical examination skills among clinical officers, a single site was designated as a training centre in HIV care and treatment. Clinical officers undergo intensive three-week training, under the supervision of experienced HIV physicians. By taking histories and examining patients together, mentors reinforce good practices and techniques, and demonstrate comprehensive history taking, physical assessment and patient management skills. Thereafter physicians at each site work closely with clinical officers to build capacity in patient management and improve quality of care provision.

Nurses qualify for targeted mentoring once they successfully complete advanced ART training. District staff members are paired with a clinical mentor, usually a registered nurse, for a minimum of two months. The goal is to acquire skills to competently manage stable patients according to established clinical protocols. Trainees conduct routine visits using data forms, simplified care algorithms, and standard operating procedures. They monitor patients' response to antiretroviral drugs, assess for toxicities, screen for treatment failure, and renew ART prescriptions. Complicated patients with moderate to severe symptoms are immediately referred to clinical officers or physicians for further assessment. Competency is assessed at the completion of the mentorship program using standardized evaluation tool in structured clinical settings. Because of the intensive nature of clinical officer and nurse training, "team leaders" for each cadre are selected on-site, with the expectation that they will continue training other members of staff during and upon completion of this clinical mentorship.

Peer educators receive intensive practical mentorship in the two weeks following didactic training. This mentorship focuses on personal appearance, self-awareness, non-verbal communication, interview techniques, cultural competency, client-centered counseling, and communication skills. Peer supervisors and nurses conduct patient counseling with new peer educators, demonstrating strategies for communication in action. Nurses train peer educators in basic clinical duties: recording basic demographic information on patients; recording weight and height; and using digital equipment to measure vital signs. Thereafter, the senior nurse manager at each clinic supervises duties performed by peer educators, assists them with complex issues, and provides psychological support. Peer educators use journals to record details of the cases they see, which are later presented to clinic nurses and peer supervisors for guidance. In bimonthly trainings, problem areas are identified and addressed through lectures and/or practical sessions.

### Continuous quality assurance

Our comprehensive continuous quality assurance program focuses on three core activities: (1) evaluation of clinical care via targeted chart reviews and monthly site reports from our electronic medical record, (2) feedback and training in areas of poor site performance, and (3) an exchange program between clinics to improve overall clinical quality. This program is coordinated by a central team of "quality assurance" (or QA) nurses and data monitors.

**Figure 2:**
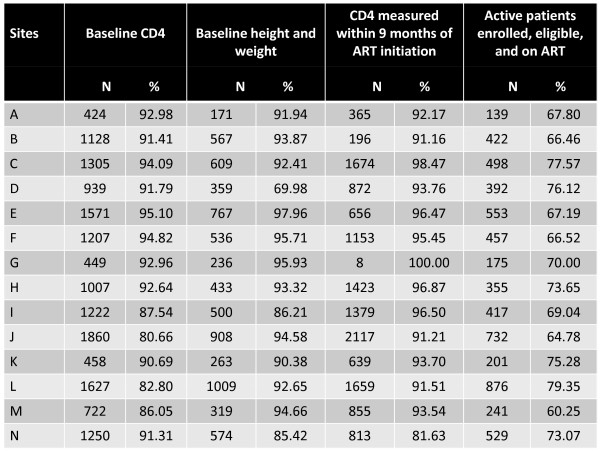
**Example of a quarterly performance report for Lusaka District HIV care and treatment programs**. ART = antiretroviral therapy.

On a quarterly basis, each program site is evaluated for clinical care performance. The establishment of a centralized electronic medical record [6] has facilitated the generation of routine site-specific performance evaluations. Several reports have been programmed to monitor clinical care. A laboratory report lists all critical values according to date and patient identification number. A treatment failure report lists individuals who meet local, non-virological criteria for treatment failure, triggering careful evaluation for a possible switch to second-line ART. QA nurses and data monitors perform targeted chart reviews and ensure that proper care was provided to patients. In cases where it was not, patients are recalled and appropriate interventions initiated. On a quarterly basis, site-specific performance reports are generated and shared with facility-based clinical staff. These reports rank site performance according to key clinical performance indicators such as: proportion of patients starting ART who have baseline laboratory results documented; proportion of patients on ART who have a repeat CD4 count ordered at the appropriate time; patient retention in care; and proportion of eligible patients receiving *Pneumocystis carinii (jiroveci) *pneumonia (PCP) prophylaxis (Figure 2). Recognizing and rewarding individual clinic performance has created a climate of healthy competition between sites. Semiannual rewards are presented to the clinics with the best performance: usually with a portion of these funds are spent on on-going clinic improvement schemes.

The second core activity is targeted trainings based on site performance. QA staff members take advantage of scheduled team meetings to provide focused training in areas previously identified through targeted file review and performance reports. The third core activity to support continuous quality improvement is a two-way exchange program, which pairs established, well-performing clinics with newer, less experienced clinics. During one-week exchange visits, staff perform peer assessments of their twin site, exchange ideas, provide feedback, and suggest areas for quality improvement. This intervention has been popular with staff as they share solutions to similar challenges that exist in all sites. Exchanges to date have involved nursing staff – in particular nurse managers – but future exchanges are planned for clinical and medical officers.

## Results

We began implementing task-shifting strategies in 2004 by using non-physician clinical officers to assess patients for eligibility for ART, to commence patients on ART, to monitor patients' outcomes, and to review for toxicities. In 2005, nurses were trained to assume expanded roles, primarily conducting triage, patient assessment, and ordering and interpreting laboratory investigations [7]. In the same year, we established our peer educator program. The number of clinical officers, nurses, and peer educators trained through our various curricula from 2005 to 2007 are shown in Table 1.

**Table 1:** Clinical officers, nurses, and peer educators trained through supported programs, January 2005 – December 2007

**Training**	**Clinical officers**	**Nurses**	**Peer educators**	**Total**
Adult HIV care and treatment	174	333	9	516

Pediatric HIV care and treatment	131	120	19	270

Adherence assessment and counseling	56	200	85	341

Triage training	-	91	-	91

Structured clinical mentorship	53	40	-	93

**Figure 3:**
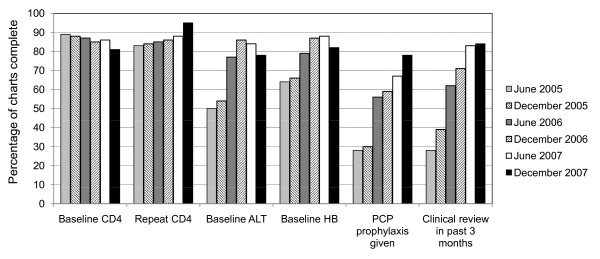
**Assessment of clinical care performance over time across the 14 Lusaka district clinics that started before June 2005**. The graphs represent the percentage of patients on antiretroviral therapy (ART) who met specific indicator of quality clinical care. ALT = alanine aminotransferase, HB = hemoglobin, PCP = *Pneumocystis carinii (jiroveci) *pneumonia

**Figure 4:**
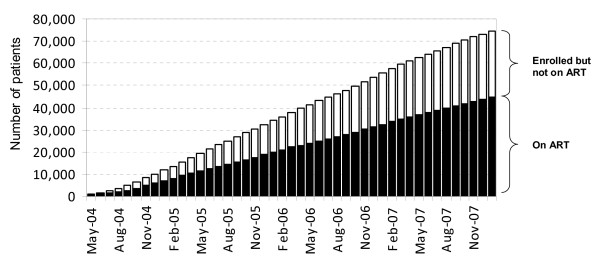
**Number of patients enrolled and commenced on antiretroviral therapy in the 14 Lusaka district clinics that started before June 2005**. This figure demonstrates that the improvements in quality care indicators were achieved in the context of rapid service roll-out. ART = antiretroviral therapy.

Although task-shifting can help alleviate the human resource shortages in Africa, one major concern has been reduced clinical care quality. To address this issue, we conducted a review of quarterly clinic performance reports in order to assess whether clinic performance had changed following introduction of task-shifting and quality assurance activities. When we examined clinical performance according to time of site establishment, we noted general improvements in several basic indicators (Figure 3) despite significant increases in clinic volumes (Figure 4). While these findings cannot be directly attributed to our task-shifting program, we find them nonetheless encouraging.

## Discussion

Like much of Africa, provision of free ART services in Lusaka has created unprecedented health systems demand that cannot be met using traditional physician-dependent models. In our setting, we have attempted to utilize every available human resource to its full potential. Our three-pronged approach of training, mentorship, and continuous quality assessment has allowed the rapid roll-out of services despite notable resource constraints. Involvement of peer educators – mostly members of the surrounding communities – has helped to reduce stigma surrounding HIV and mobilize community leaders. The quality of care has not suffered, but has instead steadily improved under a structured program of assessment and targeted training.

The primary intent of our report is to describe our task-shifting strategy and provide basic data demonstrating feasibility. We are not in a position to perform a formal effectiveness analysis of our task-shifting approach – either as a package or by its individual components – because of insurmountable methodological difficulties in this programmatic setting (e.g. on-going nature of the clinical mentorship intervention, differences in patient volume and staffing among the sites, differing provider characteristics, possibility of temporal bias). Identification of a suitable comparator arm is also difficult because almost all the facilities we sponsor have at least some degree of task-shifting in place. Our inability to measure strategy effectiveness is a recognized limitation of this report. Nonetheless, we believe there are important lessons that come out of these experiences, particularly in the area of program design.

Various studies advocate greater use of lay health care workers in response to the human resource demands of HIV care and treatment [8]. In Uganda, lay health workers have been trained to perform simple patient assessment and deliver antiretroviral medications to patients in their homes [9]. In Zambia, the use of "adherence support workers" to provide clinic-based adherence counseling has resulted in reduced patient waiting times without comprise of adherence counseling quality [10]. In our report, peer educators are only one component of the overall task-shifting strategy, making it difficult to determine their separate contribution to patient care outcomes. Nevertheless, we recognize the need for on-going supervision and performance evaluation for this newly established cadre of provider. At present, this includes daily on-site nursing supervision and site evaluations from peer supervisors on a regular basis. In addition, we are conducting follow-up research to determine patient and provider satisfaction with the peer educator strategy to objectively measure their effectiveness.

Although our model relies heavily on community members in routine care, it also promotes task-shifting within the health professions. We believe this to be a key component of our strategy's sustainability, since there are absolute limitations to the background knowledge and medical expertise that can be obtained by lay workers. Strengthening clinical abilities and experience among mid-level clinicians addresses the human resource crisis. Strategies such as this could also lead to improved job satisfaction and staff retention by reducing the risk of occupational burnout.

The clinic mentorship model described in this report is intensive and may be lengthy. On-site clinical mentorship for nurses, for example, usually lasts three months because of integration into often busy clinic flow. For this reason, we have only been only able to provide mentorship to 93 of 507 (18%) nurses and clinical officers who completed Ministry of Health-supported training workshops for adult HIV care and treatment. Our mentorship trainees were purposely chosen from senior members of staff, individuals experienced in clinical practice and teaching. The expectation is that these trainees will take on similar mentoring responsibilities for other providers on-site. We are currently developing appropriate monitoring strategies to ensure that lessons in basic clinical practices and HIV medical management are properly disseminated. This is a critical component to the sustainability of such a program, particularly as it rolls out into semi-urban and rural sites.

Strengths of this program are its focus on local capacity building and emphasis on clinical care quality rather than simple program indicators. One criticism has been its intensive use of resources; however, we believe this is justified, particularly during the early years of scale-up. The programs described in this report require trained personnel and central coordination, resources that may not be readily available in all African settings. This may be particularly true of rural settings where HIV prevalence and demand for services may be low. One possible adaptation would be the establishment of a few regional centers – likely in urban areas due to higher patient volumes – where providers can receive intensive training and then return to their primary facilities. Quality assessment could still be incorporated, though the frequency of visits may need to be reduced for feasibility.

To successfully bring this model to scale, engagement of local governments is an absolute necessity. One solution could be the integration of task-shifting into formal nursing curricula, with recognition of expanded duties via certification, legal support, and professional regulation. With such support, novel ventures such as nurse-led clinics could assist greatly with provision of necessary services [11,12]. The government of Botswana has supported one such model, by institutionalizing the nurse practitioner degree in the 1980s [13]. Similar efforts are possible in Zambia, but political and professional barriers must first be addressed at the national level [14]. The curriculum for an HIV specialty certificate – one that would allow nurses to screen patients and initiate ART – has recently been approved locally in conjunction with the University of Zambia and the Nursing Council of Zambia. In a similar vein, the Zambian Ministry of Health is working with local partners to formalize a national training program for lay health workers such as peer educators. Such an initiative would standardize clinical skills and responsibilities of these lay workers and officially integrate them into the Ministry's personnel structure.

## Conclusion

Over the short term, it is possible to expand ART services in settings of extreme health worker shortage without comprising clinical care quality. Alongside training, mentoring and continuous quality monitoring, we have also created clinic flow efficiencies, provided overtime payments to increase staffing [15], and recruited community workers and patients to support care initiatives. However, engagement with Ministries of Health is critically needed for long-term sustainable solutions: reduced provider migration (i.e. "brain drain"), expanded health care for providers with HIV, and improved working conditions for government health professionals. The human resource shortage is a critical barrier to the rapid scale of ART – and the public health benefits associated with such programs – and must to be addressed with new and innovative strategies.

## Competing interests

The authors declare that they have no competing interests.

## Authors' contributions

MM, BC, and SR designed the concept, analyzed the data, and wrote the first draft. BT and AM assisted in program design and implementation, interpreted the data, and provided substantial revisions to the manuscript. HC, JM, HM, CB, and DP provided program oversight, collected the data, and provided substantial revisions to the manuscript. JSAS contributed to the data analysis and substantially revised the manuscript. All authors approved the final version of the manuscript.

## Pre-publication history

The pre-publication history for this paper can be accessed here:


